# Identification of Driver Genes and Interaction Networks Related to Brain Metastasis in Breast Cancer Patients

**DOI:** 10.1155/2022/7631456

**Published:** 2022-01-28

**Authors:** Haojie Zhang, Xiaohong Wang, Changran Hou, Zhenlin Yang

**Affiliations:** ^1^Department of Binzhou Medical University, Yantai, Shandong 264003, China; ^2^Department of Thyroid and Breast Surgery, Binzhou Medical University Hospital, Binzhou, Shandong 256603, China

## Abstract

Brain metastasis is a common complication of breast cancer (BC); however, the interaction networks and driver genes that lead to brain metastasis in BC patients are still unknown. In this study, we employed bioinformatics analyses to discover hub genes and long noncoding RNA- (lncRNA-) protein-coding gene (PCG) networks related to BC brain metastasis (BCBM). Firstly, we screened differentially expressed PCGs and lncRNAs in normal and BCBM samples using the GSE52604 dataset. Subsequently, differentially expressed genes (DEGs) and overall interaction networks were constructed, and topological degrees were analyzed to identify potential driver genes. After identifying the hub pathogenic module by weighted gene coexpression network analysis (WGCNA), the genes in the hub module were evaluated for functional enrichment. Finally, we constructed multiple interaction networks associated with BCBM and identified seven potential driver genes, out of which MYBPC1 was the only overlapping gene in the adopted analytical methods. It is worth mentioning that we validated the prognostic value of the identified hub genes in TCGA database and evaluated the prediction ability of MYBPC1 in the GSE38057 dataset. In addition, the CIBERSORT algorithm revealed changes in the immune microenvironment. In conclusion, the driver PCGs and lncRNAs in the interaction networks can be utilized as a promising therapeutic strategy for the treatment of brain metastasis in BC patients.

## 1. Introduction

Breast cancer (BC) is the most commonly diagnosed cancer in women [1]. As with other types of cancer, metastasis poses a serious challenge in the management of BC. Metastasis is the process that causes the distant spread of BC to the liver, bones, lungs, and brain. BC is the second leading cause of metastatic encephalopathy after lung cancer [2]. Brain metastases have been found to occur in 10–16% of surviving individuals with advanced BC and as high as 30% in autopsy series [3]. Additionally, the brain is the primary location of BC metastases in 13% of patients [4]. Given the dismal prognosis associated with a BC brain metastasis (BCBM) diagnosis, knowing what motivates cancer cells to metastasize and populate the brain may be the first step toward possibly stopping metastasis and eradicating metastatic cancer cells.

Pangeni et al. analyzed the methylation status of 82 potential genes and discovered 21 commonly methylated genes in BCBM [5]. Out of the 21 genes, three genes, GALNT9, CCDC8, and BNC1, were methylated at a frequency of 55%, 73%, and 71%, respectively. While GALNT9 and BNC1 were mostly methylated and silenced in BM but not in primary BC, CCDC8 was often methylated in both. This study implies that methylation of CCDC8 occurs early in the genesis of metastatic tumors, but methylation of GALNT9 and BNC1 occurs later. Therefore, these three genes seem to be involved in the development of primary BC to brain metastases, and they may serve as important prognostic indicators, leading to novel treatment options. In addition, recent studies on the relationship between tumor cells and their microenvironment in the brain have revealed several signal transduction molecules and their regulatory mechanisms [6]. For example, in BC cells, the creation of HER2-HER3 heterodimers results in a considerable stimulation of the PI3K-Akt signaling pathway [7]. Following the above cell implantation into the brain of a mouse, increased HER3 expression was seen in a HER2-amplified BC cell line (BT-474) [8], suggesting that the HER3 activation of the PI3K-Akt pathway contributes to brain metastasis. Till today, multiple studies have focused on identifying BCBM driver genes and the possible causes for brain metastasis in BC patients. However, the fundamental processes behind metastatic development in the central nervous system (CNS) are unclear, and the regulatory relationship between the important protein-coding genes (PCGs) and the long noncoding RNAs (lncRNAs) has not been clearly explained. The purpose of this research is to discover the key driver genes and interaction networks associated with BCBM in order to deduce the biological processes behind brain metastasis and to offer a theoretical foundation for future investigations.

## 2. Materials and Methods

### 2.1. Public Datasets

GSE52604 and GSE38057 datasets, containing the transcriptomic data and clinical information of the patients, were obtained from the GEO database. The datasets were annotated using the GPL6480/GPL5826 platform files. The GSE52604 dataset included 35 BC samples with brain metastasis, ten nonneoplastic brain samples, and ten nonneoplastic breast samples. In this study, bioinformatics analyses were performed for the GSE52604 dataset. The GSE38057 dataset was used to verify the predictive ability of the screened hub genes in the GSE52604 dataset. In addition, we verified the expression of mRNA and protein corresponding to the identified hub genes in TCGA and HPA databases, respectively.

### 2.2. Differential Expression Analysis

The differentially expressed genes (DEGs) in the BCBM and normal samples were screened using the limma package, and ∣log2 fold change (FC) | >3.5 and the adjusted *P* value of <0.05 were selected as cutoff criteria.

### 2.3. Construction of Interaction Networks

Spearman's correlation coefficients were calculated based on the expressions of PCGs and lncRNAs to construct a lncRNA-PCG interaction network for BCBM (∣*r*  | ≥0.5, *P* < 0.05). After this, the network was visualized, and the node degrees were analyzed to identify driver genes.

### 2.4. Weighted Correlation Network Analysis (WGCNA) of Coexpressed Genes

WGCNA was performed with a gene expression matrix of 1,429 possible driver genes (cut height = 60, with an optimal soft threshold of six). According to the soft threshold, the relationship matrix was converted into an adjacency matrix and then into a topological overlap matrix (TOM) for mean linkage hierarchical clustering. The related modules were classified according to the TOM, with the number of genes in each module not less than 50. The similar modules were merged based on the gene module shearing height of 0.25. Finally, the correlation of the merged modules with clinical traits was determined using Pearson's correlation.

### 2.5. Functional Enrichment Analysis

Gene ontology (GO) enrichment analysis was utilized to search for comprehensive information on the large-scale genetic data. In addition, Kyoto Encyclopedia of Genes and Genomes (KEGG) pathway enrichment analysis was employed to understand the biological mechanisms and functions. Visualization of GO and KEGG analyses was performed by utilizing the GOplot package.

### 2.6. Immune Cell Infiltration Analysis

The CIBERSORT algorithm was used to explore the proportion of different types of immune cells according to the expression level of immune cell-related genes [9].

### 2.7. Statistical Analysis

All statistical analyses were performed using the R software (v.4.0.1). Detailed statistical methods for RNA-sequence processing are mentioned in the above section.

## 3. Results

### 3.1. Differential Expression Analysis

**Figure 1:**
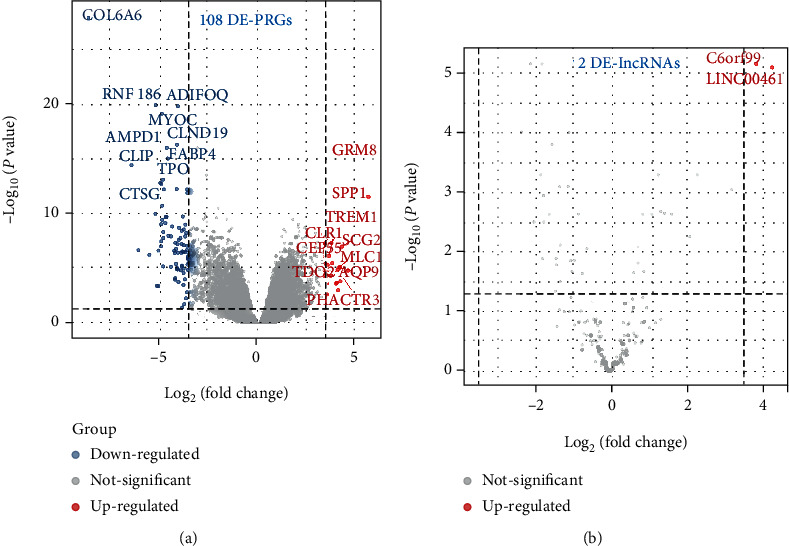
Identification of differentially expressed PCGs and lncRNAs. (a) 108 DE-PCGs in the volcano plot. (b) 2 DE-lncRNAs in the volcano plot. Blue dots represent downregulated genes, and red dots represent upregulated genes.

We explored the differential expression of PCGs and lncRNAs in 35 BCBM and ten normal samples. A total of 108 differentially expressed PCGs (Figure 1(a)) and two differentially expressed lncRNAs (Figure 1(b)) were identified. Overall, our data identified a gene panel responsible for the occurrence of BCBM.

### 3.2. Construction of Interaction Networks Related to BCBM

**Figure 2:**
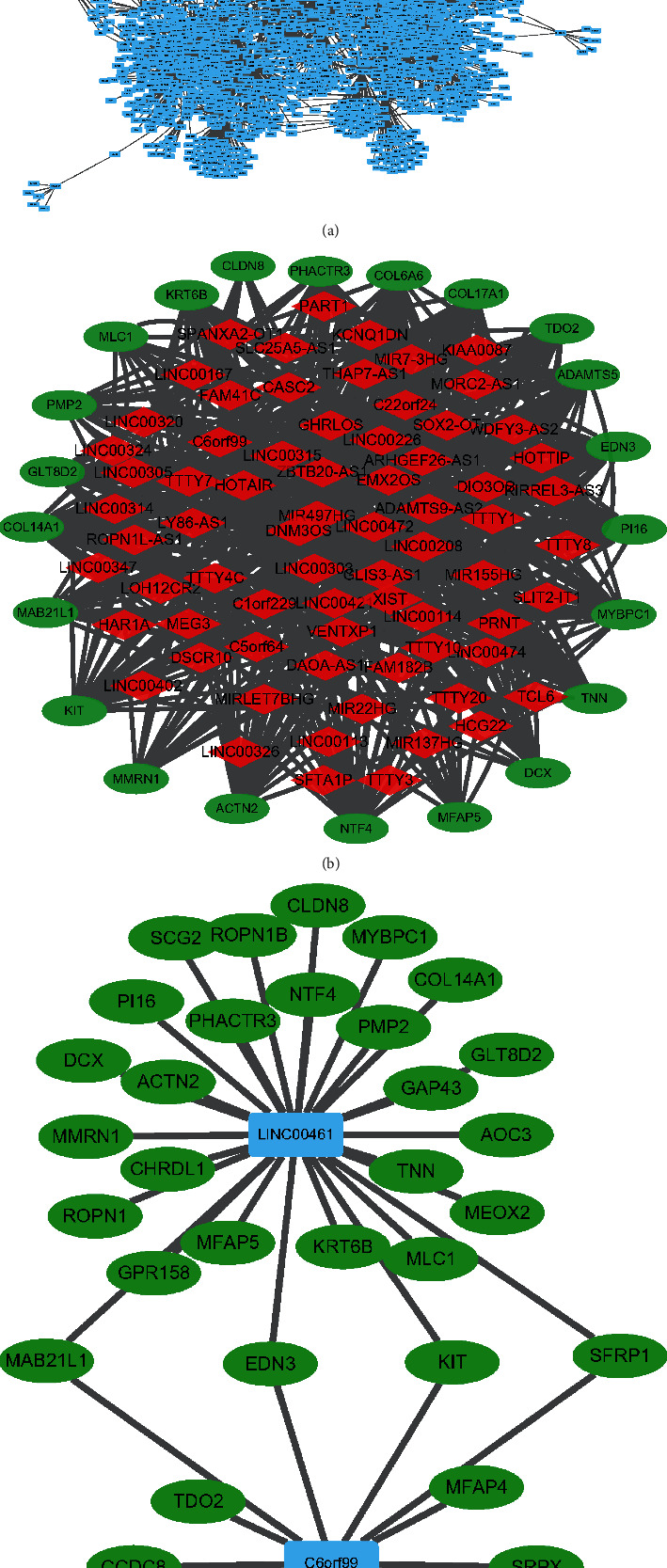
Interaction networks related to BCBM. (a) All landscape of PCGs-lncRNAs. (b) Driving interaction networks. (c) Interaction networks of DE-PCGs and DE-lncRNAs.

We calculated the correlation coefficients of PCG and lncRNA coexpression in 35 BCBM samples. Finally, we identified 18,810 pairs of PCGs-lncRNAs (Supplementary file 1), visualized the network, and analyzed the topological degree of each gene (Figure 2(a)). To identify the hub genes in the network, we set the threshold to three and screened 1,429 potential driver genes. Subsequently, we calculated the correlation coefficients for 1,429 genes again and identified a driver network consisting of 584 pairs of PCGs-lncRNAs (Figure 2(b)). Meanwhile, regulatory interactions between 108 differentially expressed PCGs and two differentially expressed lncRNAs (C6orf99 and LINC00461) were also visualized on Cytoscape (Figure 2(c)). Finally, we overlapped the potential driving genes and 108 differentially expressed PCGs, thereby identifying five hub PCGs, including MLC1, MYBPC1, PHACTR3, GAP43, and PMP2. Thus, our data showed three visual networks (pairs of PCGs-lncRNAs) and five potential hub PCGs driving the development of BCBM.

### 3.3. Validation of Hub PCG Expression

**Figure 3:**
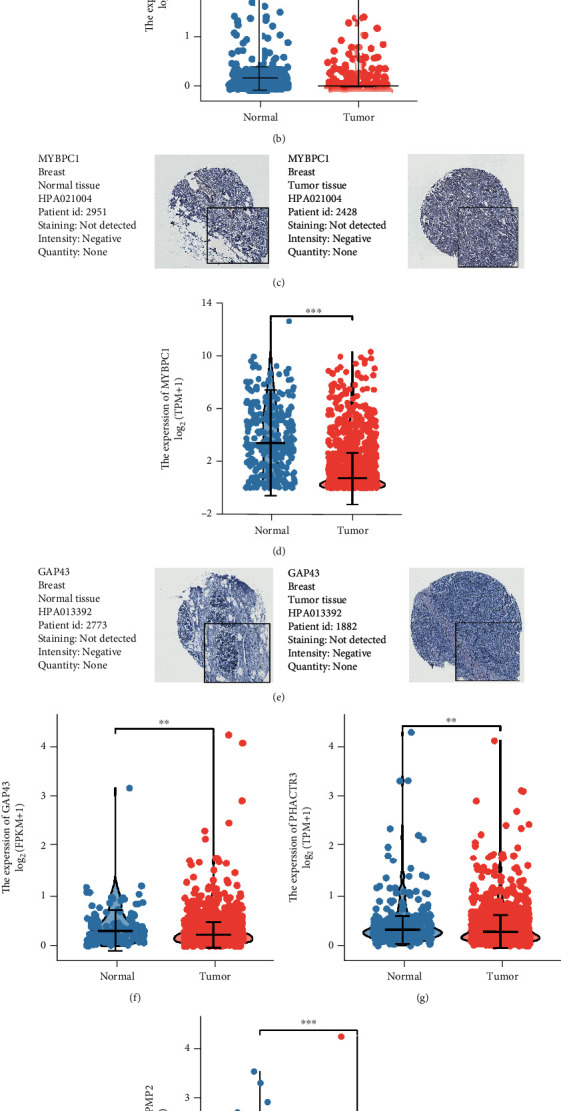
Validation of mRNA and protein. (a) IHC sections of MLC1 in different tissue samples. (b) MLC1 mRNA expression in different tissue samples. (c) IHC sections of MYBPC1 in different tissue samples. (d) MYBPC1 mRNA expression in different tissue samples. (e) IHC sections of GAP43 in different tissue samples. (f) GAP43 mRNA expression in different tissue samples. (g) PHACTR3 mRNA expression in different tissue samples. (h) PMP2 mRNA expression in different tissue samples.

We searched the HPA database to verify the hub PCG expression and additionally combined the GTEx and TCGA databases to explore the mRNA expression. Not surprisingly, mRNA expression analysis demonstrated that the hub PCGs were significantly differentially expressed in different tissues (Figures 3(b), 3(d), and 3(f)–3(h)). Unfortunately, we could not verify the protein expression of PHACTR3 and PMP2 in the HPA database; moreover, only the protein expression of the MLC1 gene exhibited variations in different tissues (Figures 3(a), 3(c), and 3(e)). Therefore, we conjectured that epigenetic modification might be involved in the hub PCGs. Taken together, our data revealed the landscape of expression of the MLC1, MYBPC1, PHACTR3, GAP43, and PMP2 genes.

### 3.4. WGCNA

**Figure 4:**
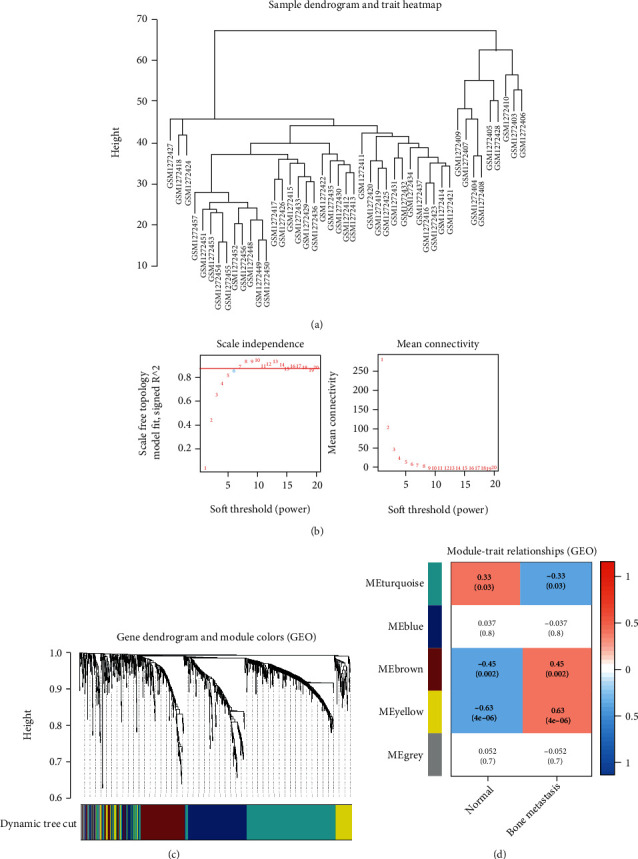
WGCNA. (a) Sample clustering. (b) Optimal soft threshold selection. (c) Clustering different modules. (d) Correlation between modules and occurrence of brain metastases.

WGCNA was performed with a gene expression matrix of 1,429 possible driver PCGs (Figures 4(a) and 4(b)). We explored the following five modules from the gene expression profile: blue (283 genes), brown (229 genes), grey (50 genes), turquoise (457 genes), and yellow modules (104 genes) (Figures 4(c) and 4(d)). Further analysis revealed that the genes in the yellow module were associated with brain metastasis of BC (*r* = 0.63). Thus, our data revealed the potential gene set (yellow module in WGCNA) that may be associated with the occurrence of BCBM.

### 3.5. Functional Enrichment Analysis

**Figure 5:**
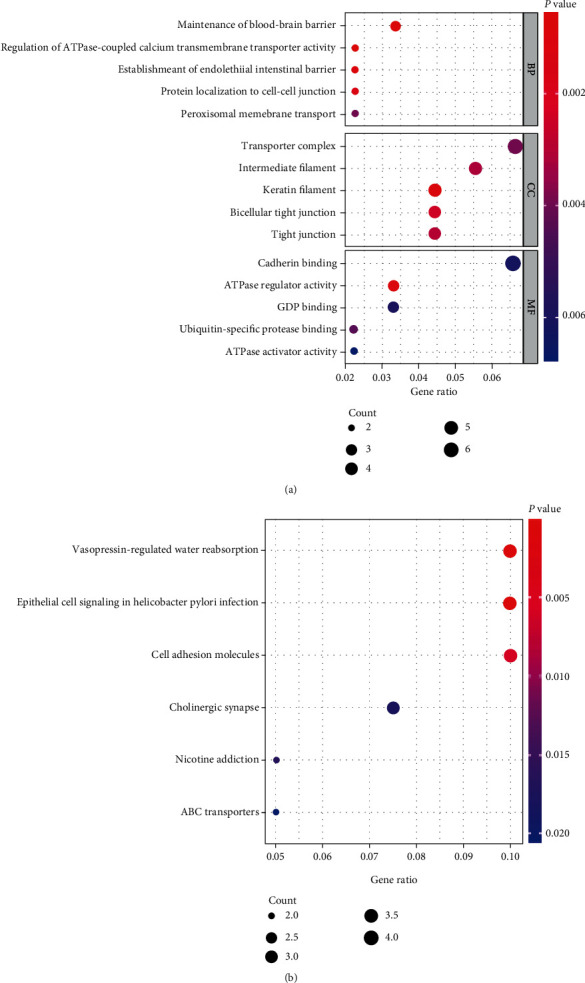
Functional enrichment analysis. (a) GO analysis. (b) KEGG analysis.

The yellow module consisted of 104 genes, including MYBPC1, one of the hub driver genes. GO and KEGG analyses were employed for functional enrichment of genes in the yellow module. The results of GO analysis indicated that the above genes might be involved in the maintenance of the blood-brain barrier (BBB), regulation of ATPase-coupled calcium transmembrane transporter activity, and establishment of the endothelial intestinal barrier (Figure 5(a)). Based on the KEGG analysis, we identified important functional pathways, which suggested that these genes in the yellow module may be associated with vasopressin-regulated water reabsorption, epithelial cell signaling in Helicobacter pylori infection, cell adhesion molecules, nicotine addiction, cholinergic synapse, and ABC transporters (Figure 5(b)). Thus, our data identified some crucial biological processes and pathways responsible for the occurrence of BCBM.

### 3.6. Interaction Network in the Yellow Module

**Figure 6:**
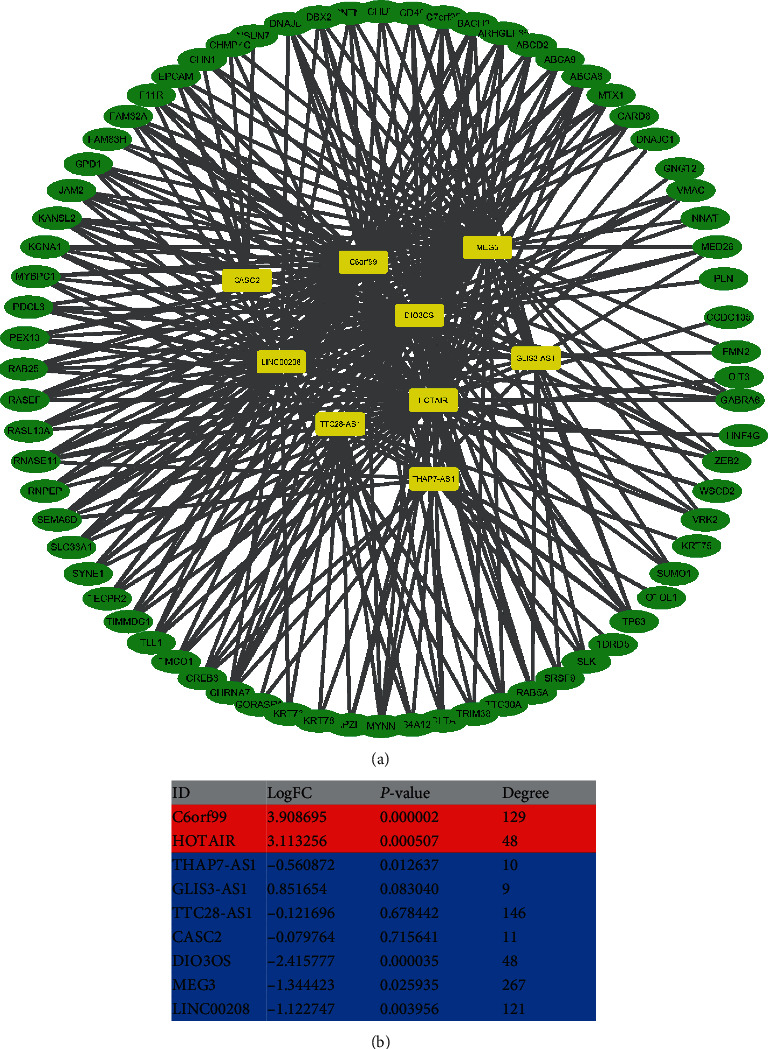
Interaction network of the hub module. (a) Interaction network. The yellow squares represent lncRNAs, and the blue squares represent PCGs. (b) The logFC and topology degree of 9 lncRNAs in the hub module.

The visual network revealed the subtle interaction between lncRNAs and PCGs in the yellow module (Figure 6(a)). We analyzed the topological degree and differential expression status of each lncRNA (Figure 6(b)). The results demonstrated that two lncRNAs (C6orf99 and HOTAIR) were significantly upregulated in BCBM (logFC = 3.908695 and 3.113256; degree = 129 and 48). In addition, we conducted correlation analysis on 94 PCGs and nine lncRNAs in the yellow module (Table S1), demonstrating that C6orf99 was correlated with 49 PCGs, CASC2 with four PCGs, DIO3OS with 52 PCGs, GLIS3-AS1 with 17 PCGs, HOTAIR with 48 PCGs, LINC00208 with four PCGs, MEG3 with 51 PCGs, ThAP7-AS1 with 13 PCGs, and TTC28-AS1 with 14 PCGs.

### 3.7. Prognostic Analysis and External Validation

**Figure 7:**
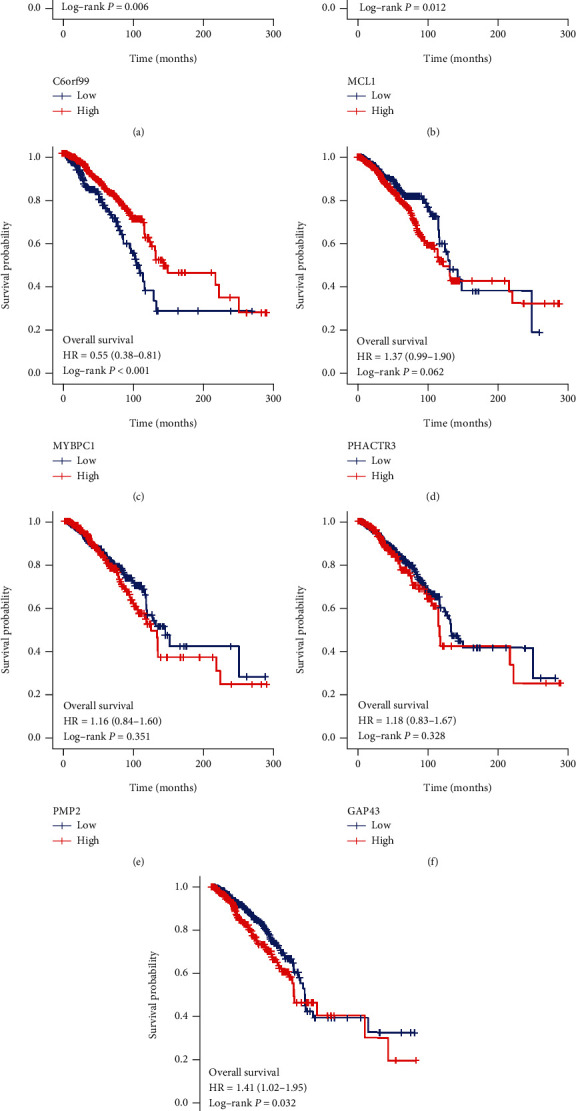
Kaplan-Meier analysis in TCGA database. Kaplan-Meier analysis of C6orf99 (a), MLC1 (b), MYBPC1 (c), PHACTR3 (d), PMP2 (e), GAP43 (f), and LINC00461 (g).

**Figure 8:**
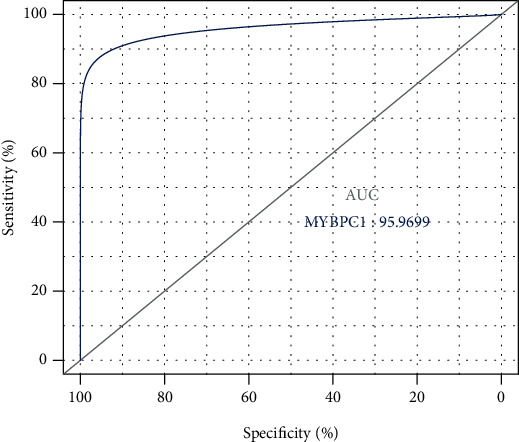
Validation of predictive ability (MYBPC1).

The Kaplan-Meier analysis indicated that the identified hub genes, except for PHACTR3, PMP2, and GAP43, were associated with the overall survival (OS) of BC patients (Figure 7). To further validate the clinical application of hub genes in the assessment of BCBM, we performed external validation of MYBPC1. The ROC analysis of the GSE38057 dataset indicated that MYBPC1 could well confirm the possibility of brain metastasis in BC patients (AUC = 0.959), as shown in Figure 8.

### 3.8. Immune Cell Infiltration Analysis

**Figure 9:**
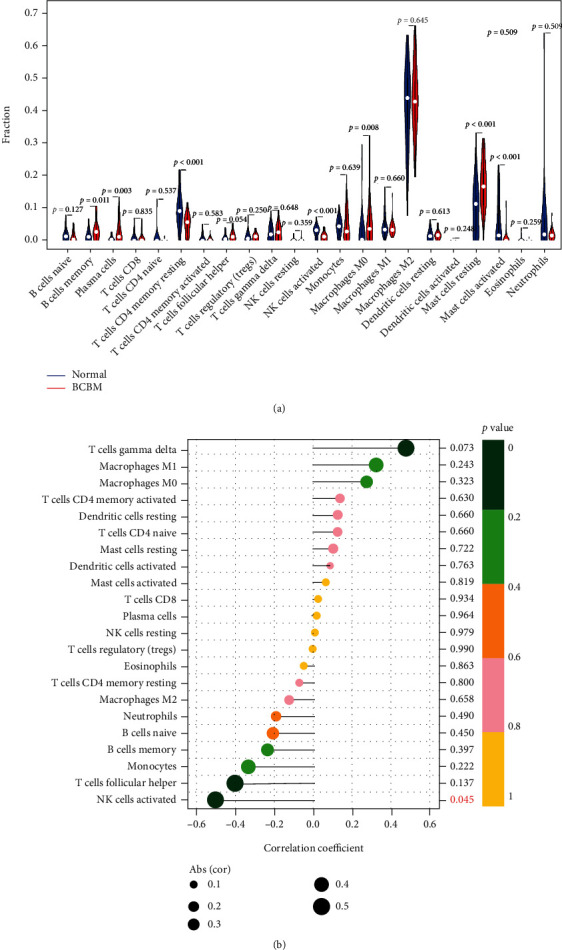
Immune cell infiltration analysis. (a) Differences in immune cell content. (b) Pearson's correlation analysis of MYBPC1 and immune cells.

The Wilcoxon test showed higher B cell memory, plasma cells, macrophage M0, and mast cells resting in BCBM samples. In normal tissues, only T cell CD4 memory resting and NK cells activated have higher contents (Figure 9(a)). In BCBM tissues, the correlation between 22 types of immune cells and MYBPC1 was analyzed, and results showed that MYBPC1 expression was positively correlated with NK cell content (Figure 9(b)).

## 4. Discussion

Presently, specific therapies targeting brain metastases in BC patients are not established, making their prognosis even more dismal. Therefore, identifying the driver genes and interaction networks might provide the groundwork for treating the illness [2]. Hence, in the present study, we compared different samples in the GSE52604 dataset and identified 108 differentially expressed PCGs and two differentially expressed lncRNAs (C6orf99 and LINC00461) in total. Subsequently, we identified 18,810 pairs of PCGs-lncRNAs and analyzed the topological degree of each gene to screen 1,429 potential driver genes. Similarly, we also identified a driving network, including 584 pairs of PCGs-lncRNAs and regulatory interactions between 108 DEGs. We overlapped the potential driver genes and 108 differentially expressed PCGs, thereby identifying five hub PCGs, including MLC1, MYBPC1, PHACTR3, GAP43, and PMP2. Meanwhile, we searched the HPA, TCGA, and GTEx databases to verify the expression and prognostic abilities of the identified hub PCGs. In addition, we utilized WGCNA to screen a hub gene set, including nine lncRNAs and 95 PCGs, most notably, MYBPC1, one of the hub driver genes. Finally, MYBPC1 was considered a candidate predictor in BCBM. Meanwhile, Pearson's results showed that MYBPC1 expression was positively correlated with NK cell content. The MYBPC1 gene, which encodes a slow skeleton isomer (sMyBP-C), has received much attention recently [10]. Unfortunately, there are no in vitro studies on the role of the MYBPC1 gene in tumor metastasis. A study conducted on zebrafish with MYBPC1 deficiency demonstrated severe ventral body curvature, decreased motility, and early death, as well as defective myopod development and decreased myogenic fiber counts [11]. Thus, it can be concluded that the MYBPC1 gene leads to severe and lethal myopathies [12]. However, the exact mechanism of MYBPC1 in BCBM needs to be further explored.

Among the identified hub driver lncRNAs, C6orf99 has been used in the construction of several prediction models, such as BC [13] and liver cancer [14]. Recent studies have shown LINC00461 to inhibit the epithelial-mesenchymal transition (EMT) of non-small-cell lung cancer cells [15]. Also, higher levels of LINC00461 are observed in BC tissues and cells, and inhibiting it may decrease vimentin expression while increasing E-cadherin expression [16]. Additionally, studies indicated that silencing LINC00461 dramatically decreased multiple myeloma cell growth and increased apoptosis [17]. Nevertheless, the functional details of the abovementioned lncRNAs in BCBM remain elusive. Functional enrichment analysis demonstrated that the occurrence of BCBM may be related to the maintenance of BBB, cell adhesion molecules, and the establishment of the endothelial intestinal barrier. As reported in previous studies, cell adhesion molecules are thought to be important regulators of the development of distant metastasis in BC, such as cadherins, selectins, and integrins [18]. Meanwhile, disruption of the BBB has been observed in BC patients who developed BCBM. The signaling pathways and processes that mediate the early steps in the extravasation of breast tumor cells across the brain microvascular endothelium have been systematically described in a review [19]. Therefore, we can combine the abovementioned biological processes, driver genes, and interaction networks to explore the real mechanism of BCBM.

This study has a few limitations. First, the study utilized the GSE52604 dataset for analysis and the GSE38057 dataset for validation; however, in vitro or in vivo assays were not performed. Second, our sample size was small, and no validation was performed on the native cohort. Finally, our interaction networks focused on the relationship between lncRNAs and PCGs only, without an in-depth exploration of transcription factors (TFs), microRNAs, etc.

## 5. Conclusion

In this study, we identified seven driver genes, three interaction networks, and underlying biological processes related to BCBM by using a series of bioinformatics analyses. In conclusion, targeting driver PCGs and lncRNAs in the interaction networks may be a promising therapeutic strategy for the treatment of brain metastasis in BC patients.

## Data Availability

The following information was supplied regarding data availability: data is available at TCGA (https://portal.gdc.cancer.gov/) and GEO databases (https://www.ncbi.nlm.nih.gov/geo/).

## Supplementary Materials

Supplementary 1 Supplementary files 1: 18,810 pairs of PCGs-lncRNAs.

Supplementary 2 Table S1: correlation analysis on 94 PCGs and 9 lncRNAs.

## References

[1] Ullah M. F. (2019). Breast cancer: current perspectives on the disease status. *Advances in Experimental Medicine and Biology*.

[2] Lin N. U. (2013). Breast cancer brain metastases: new directions in systemic therapy. *Ecancermedicalscience*.

[3] Arslan U. Y., Oksuzoglu B., Aksoy S. (2011). Breast cancer subtypes and outcomes of central nervous system metastases. *Breast*.

[4] Altundag K., Bondy M. L., Mirza N. Q. (2007). Clinicopathologic characteristics and prognostic factors in 420 metastatic breast cancer patients with central nervous system metastasis. *Cancer*.

[5] Pangeni R. P., Channathodiyil P., Huen D. S. (2015). The GALNT9, BNC1 and CCDC8 genes are frequently epigenetically dysregulated in breast tumours that metastasise to the brain. *Clinical Epigenetics*.

[6] Hosonaga M., Saya H., Arima Y. (2020). Molecular and cellular mechanisms underlying brain metastasis of breast cancer. *Cancer Metastasis Reviews*.

[7] Lee-Hoeflich S. T., Crocker L., Yao E. (2008). A central role for HER3 in HER2-amplified breast cancer: implications for targeted therapy. *Cancer Research*.

[8] Kodack D. P., Chung E., Yamashita H. (2012). Combined targeting of HER2 and VEGFR2 for effective treatment of HER2-amplified breast cancer brain metastases. *Proceedings of the National Academy of Sciences of the United States of America*.

[9] Chen B., Khodadoust M. S., Liu C. L., Newman A. M., Alizadeh A. A. (2018). Profiling tumor infiltrating immune cells with CIBERSORT. *Methods in Molecular Biology*.

[10] Harris S. P., Lyons R. G., Bezold K. L. (2011). In the thick of it: HCM-causing mutations in myosin binding proteins of the thick filament. *Circulation Research*.

[11] Ha K., Buchan J. G., Alvarado D. M. (2013). MYBPC1 mutations impair skeletal muscle function in zebrafish models of arthrogryposis. *Human Molecular Genetics*.

[12] Geist J., Kontrogianni-Konstantopoulos A. (2016). MYBPC1, an emerging myopathic gene: what we know and what we need to learn. *Frontiers in Physiology*.

[13] Xiao R., Yang M., Tan Y., Ding R., Li D. (2021). Identification of five immune-related lncRNAs predicting survival and tumor microenvironment characteristics in breast cancer. *Computational and Mathematical Methods in Medicine*.

[14] Li X., Jin F., Li Y. (2021). A novel autophagy-related lncRNA prognostic risk model for breast cancer. *Journal of Cellular and Molecular Medicine*.

[15] Li C.-F., Chen J.-Y., Ho Y.-H. (2019). Snail-induced claudin-11 prompts collective migration for tumour progression. *Nature Cell Biology*.

[16] Dong L., Qian J., Chen F., Fan Y., Long J. (2019). LINC00461 promotes cell migration and invasion in breast cancer through miR-30a-5p/integrin *β*3 axis. *Journal of Cellular Biochemistry*.

[17] Deng M., Yuan H., Liu S., Hu Z., Xiao H. (2019). Exosome-transmitted LINC00461 promotes multiple myeloma cell proliferation and suppresses apoptosis by modulating microRNA/BCL-2 expression. *Cytotherapy*.

[18] Li D.-M., Feng Y.-M. (2011). Signaling mechanism of cell adhesion molecules in breast cancer metastasis: potential therapeutic targets. *Breast Cancer Research and Treatment*.

[19] Arshad F., Wang L., Sy C., Avraham S., Avraham H. K. (2011). Blood-brain barrier integrity and breast cancer metastasis to the brain. *Pathology Research International*.

