# Supplementary material for: Identification of Driver Genes and Interaction Networks Related to Brain Metastasis in Breast Cancer Patients
Source: Dis Markers. 2022 Jan 28;2022:7631456. doi: 10.1155/2022/7631456 (PMC8817105; doi:10.1155/2022/7631456)
Supplement: Supplementary 2 — Table S1: correlation analysis on 94 PCGs and 9 lncRNAs. [file 7631456.f2.docx]

**Table S1. Correlation analysis on 94 PCGs and 9 lncRNAs.**

| **Gene** | **lncRNA** | **cor** | **P-value** |
| --- | --- | --- | --- |
| ABCA8 | C6orf99 | -0.6240 | 0.0000 |
| ABCA9 | C6orf99 | -0.7479 | 0.0000 |
| ABCD2 | C6orf99 | -0.3614 | 0.0147 |
| ARHGEF35 | C6orf99 | 0.3511 | 0.0180 |
| BACH2 | C6orf99 | -0.6468 | 0.0000 |
| C7orf25 | C6orf99 | 0.4239 | 0.0037 |
| CARD6 | C6orf99 | -0.6379 | 0.0000 |
| CD46 | C6orf99 | 0.5727 | 0.0000 |
| CHRNA7 | C6orf99 | -0.4663 | 0.0012 |
| CHUK | C6orf99 | 0.3414 | 0.0217 |
| CIITA | C6orf99 | -0.3758 | 0.0110 |
| CNTN1 | C6orf99 | -0.4342 | 0.0029 |
| CREB3 | C6orf99 | 0.3573 | 0.0160 |
| DBX2 | C6orf99 | -0.6099 | 0.0000 |
| DNAJB1 | C6orf99 | 0.4248 | 0.0036 |
| DNAJC1 | C6orf99 | 0.4610 | 0.0014 |
| EPCAM | C6orf99 | 0.5124 | 0.0003 |
| F11R | C6orf99 | 0.6437 | 0.0000 |
| FAM32A | C6orf99 | 0.3726 | 0.0117 |
| FAM83H | C6orf99 | 0.3116 | 0.0372 |
| GABRA6 | C6orf99 | -0.4168 | 0.0044 |
| GPD1 | C6orf99 | -0.5947 | 0.0000 |
| JAM2 | C6orf99 | -0.5468 | 0.0001 |
| KANSL2 | C6orf99 | 0.5713 | 0.0000 |
| KCNA1 | C6orf99 | -0.4511 | 0.0019 |
| MTX1 | C6orf99 | 0.5468 | 0.0001 |
| MYBPC1 | C6orf99 | -0.5451 | 0.0001 |
| MYNN | C6orf99 | 0.4357 | 0.0028 |
| PDCL3 | C6orf99 | 0.5296 | 0.0002 |
| PEX13 | C6orf99 | 0.6107 | 0.0000 |
| RAB25 | C6orf99 | 0.6620 | 0.0000 |
| RAB5A | C6orf99 | 0.4245 | 0.0037 |
| RASEF | C6orf99 | 0.5798 | 0.0000 |
| RASL10A | C6orf99 | -0.6488 | 0.0000 |
| RNASE11 | C6orf99 | -0.4933 | 0.0006 |
| RNPEP | C6orf99 | 0.6277 | 0.0000 |
| SEMA6D | C6orf99 | -0.6183 | 0.0000 |
| SLC33A1 | C6orf99 | 0.4432 | 0.0023 |
| SRSF9 | C6orf99 | 0.5094 | 0.0004 |
| SUMO1 | C6orf99 | 0.3909 | 0.0079 |
| SYNE1 | C6orf99 | -0.4413 | 0.0024 |
| TECPR2 | C6orf99 | -0.6223 | 0.0000 |
| TIMMDC1 | C6orf99 | 0.5065 | 0.0004 |
| TLL1 | C6orf99 | -0.6497 | 0.0000 |
| TMCO1 | C6orf99 | 0.6289 | 0.0000 |
| TP63 | C6orf99 | -0.6466 | 0.0000 |
| TRIM38 | C6orf99 | 0.3140 | 0.0357 |
| TTC30A | C6orf99 | 0.6178 | 0.0000 |
| ZEB2 | C6orf99 | -0.5557 | 0.0001 |
| CHMP4C | CASC2 | 0.3913 | 0.0079 |
| CHN1 | CASC2 | -0.3238 | 0.0300 |
| EPCAM | CASC2 | 0.3235 | 0.0302 |
| NSUN7 | CASC2 | 0.3358 | 0.0241 |
| ABCA8 | DIO3OS | 0.6698 | 0.0000 |
| ABCA9 | DIO3OS | 0.6043 | 0.0000 |
| ABCD2 | DIO3OS | 0.3269 | 0.0284 |
| ARHGEF35 | DIO3OS | -0.3495 | 0.0186 |
| BACH2 | DIO3OS | 0.6505 | 0.0000 |
| C7orf25 | DIO3OS | -0.3486 | 0.0189 |
| CARD6 | DIO3OS | 0.4272 | 0.0034 |
| CD46 | DIO3OS | -0.3573 | 0.0160 |
| CHRNA7 | DIO3OS | 0.5028 | 0.0004 |
| CHUK | DIO3OS | -0.4493 | 0.0020 |
| CIITA | DIO3OS | 0.3817 | 0.0097 |
| CNTN1 | DIO3OS | 0.4626 | 0.0014 |
| CREB3 | DIO3OS | -0.3817 | 0.0097 |
| DBX2 | DIO3OS | 0.5740 | 0.0000 |
| DNAJB1 | DIO3OS | -0.6726 | 0.0000 |
| EPCAM | DIO3OS | -0.3020 | 0.0437 |
| F11R | DIO3OS | -0.5005 | 0.0005 |
| FAM32A | DIO3OS | -0.6370 | 0.0000 |
| GABRA6 | DIO3OS | 0.4772 | 0.0009 |
| GPD1 | DIO3OS | 0.4957 | 0.0005 |
| JAM2 | DIO3OS | 0.6692 | 0.0000 |
| KANSL2 | DIO3OS | -0.4677 | 0.0012 |
| KCNA1 | DIO3OS | 0.3766 | 0.0108 |
| MED26 | DIO3OS | -0.5274 | 0.0002 |
| MTX1 | DIO3OS | -0.3574 | 0.0159 |
| MYBPC1 | DIO3OS | 0.5680 | 0.0000 |
| MYNN | DIO3OS | -0.4014 | 0.0063 |
| NNAT | DIO3OS | 0.4105 | 0.0051 |
| PDCL3 | DIO3OS | -0.5022 | 0.0004 |
| PEX13 | DIO3OS | -0.5527 | 0.0001 |
| PLN | DIO3OS | 0.3171 | 0.0338 |
| RAB25 | DIO3OS | -0.4377 | 0.0026 |
| RAB5A | DIO3OS | -0.5675 | 0.0000 |
| RASEF | DIO3OS | -0.3045 | 0.0420 |
| RASL10A | DIO3OS | 0.5744 | 0.0000 |
| RNASE11 | DIO3OS | 0.5531 | 0.0001 |
| RNPEP | DIO3OS | -0.6032 | 0.0000 |
| SEMA6D | DIO3OS | 0.6428 | 0.0000 |
| SLC33A1 | DIO3OS | -0.3770 | 0.0107 |
| SLK | DIO3OS | -0.3194 | 0.0324 |
| SRSF9 | DIO3OS | -0.4663 | 0.0012 |
| SUMO1 | DIO3OS | -0.4392 | 0.0025 |
| SYNE1 | DIO3OS | 0.4095 | 0.0052 |
| TECPR2 | DIO3OS | 0.5624 | 0.0001 |
| TIMMDC1 | DIO3OS | -0.5531 | 0.0001 |
| TLL1 | DIO3OS | 0.6693 | 0.0000 |
| TMCO1 | DIO3OS | -0.3114 | 0.0373 |
| TP63 | DIO3OS | 0.6961 | 0.0000 |
| TTC30A | DIO3OS | -0.3513 | 0.0180 |
| VMAC | DIO3OS | -0.3163 | 0.0343 |
| VRK2 | DIO3OS | -0.3090 | 0.0389 |
| ZEB2 | DIO3OS | 0.5505 | 0.0001 |
| C7orf25 | GLIS3-AS1 | 0.4227 | 0.0038 |
| CD46 | GLIS3-AS1 | 0.4316 | 0.0031 |
| CHN1 | GLIS3-AS1 | -0.3306 | 0.0266 |
| CHRNA7 | GLIS3-AS1 | -0.3762 | 0.0109 |
| CHUK | GLIS3-AS1 | 0.3018 | 0.0439 |
| FAM32A | GLIS3-AS1 | 0.3166 | 0.0341 |
| JAM2 | GLIS3-AS1 | -0.4188 | 0.0042 |
| KANSL2 | GLIS3-AS1 | 0.3554 | 0.0166 |
| MED26 | GLIS3-AS1 | 0.4197 | 0.0041 |
| PDCL3 | GLIS3-AS1 | 0.4039 | 0.0059 |
| RAB5A | GLIS3-AS1 | 0.4694 | 0.0011 |
| RNASE11 | GLIS3-AS1 | -0.3690 | 0.0126 |
| SLC33A1 | GLIS3-AS1 | 0.3914 | 0.0078 |
| SRSF9 | GLIS3-AS1 | 0.3589 | 0.0155 |
| SUMO1 | GLIS3-AS1 | 0.4519 | 0.0018 |
| TP63 | GLIS3-AS1 | -0.4278 | 0.0034 |
| ZEB2 | GLIS3-AS1 | -0.3503 | 0.0183 |
| ABCA8 | HOTAIR | -0.4180 | 0.0043 |
| ABCA9 | HOTAIR | -0.6161 | 0.0000 |
| ABCD2 | HOTAIR | -0.3788 | 0.0103 |
| ARHGEF35 | HOTAIR | 0.3400 | 0.0223 |
| BACH2 | HOTAIR | -0.4486 | 0.0020 |
| C7orf25 | HOTAIR | 0.3357 | 0.0242 |
| CARD6 | HOTAIR | -0.5900 | 0.0000 |
| CD46 | HOTAIR | 0.3980 | 0.0068 |
| CIITA | HOTAIR | -0.3091 | 0.0388 |
| CNTN1 | HOTAIR | -0.3257 | 0.0290 |
| CREB3 | HOTAIR | 0.4137 | 0.0047 |
| DBX2 | HOTAIR | -0.5293 | 0.0002 |
| DNAJB1 | HOTAIR | 0.4799 | 0.0009 |
| DNAJC1 | HOTAIR | 0.5297 | 0.0002 |
| EPCAM | HOTAIR | 0.4452 | 0.0022 |
| F11R | HOTAIR | 0.5763 | 0.0000 |
| FAM32A | HOTAIR | 0.4659 | 0.0013 |
| GABRA6 | HOTAIR | -0.4221 | 0.0039 |
| GPD1 | HOTAIR | -0.3870 | 0.0086 |
| JAM2 | HOTAIR | -0.3489 | 0.0188 |
| KANSL2 | HOTAIR | 0.4292 | 0.0033 |
| KCNA1 | HOTAIR | -0.4712 | 0.0011 |
| KRT78 | HOTAIR | -0.3508 | 0.0181 |
| MPZL1 | HOTAIR | 0.4374 | 0.0027 |
| MTX1 | HOTAIR | 0.5556 | 0.0001 |
| MYNN | HOTAIR | 0.4105 | 0.0051 |
| PDCL3 | HOTAIR | 0.3473 | 0.0194 |
| PEX13 | HOTAIR | 0.4178 | 0.0043 |
| RAB25 | HOTAIR | 0.5818 | 0.0000 |
| RAB5A | HOTAIR | 0.3563 | 0.0163 |
| RASEF | HOTAIR | 0.5972 | 0.0000 |
| RASL10A | HOTAIR | -0.4132 | 0.0048 |
| RNASE11 | HOTAIR | -0.3626 | 0.0144 |
| RNPEP | HOTAIR | 0.5833 | 0.0000 |
| SEMA6D | HOTAIR | -0.4425 | 0.0023 |
| SLC33A1 | HOTAIR | 0.3133 | 0.0361 |
| SRSF9 | HOTAIR | 0.3321 | 0.0258 |
| SUMO1 | HOTAIR | 0.3029 | 0.0432 |
| SYNE1 | HOTAIR | -0.4502 | 0.0019 |
| TECPR2 | HOTAIR | -0.3405 | 0.0221 |
| TIMMDC1 | HOTAIR | 0.3802 | 0.0100 |
| TLL1 | HOTAIR | -0.5110 | 0.0003 |
| TMCO1 | HOTAIR | 0.5637 | 0.0001 |
| TP63 | HOTAIR | -0.4947 | 0.0006 |
| TTC30A | HOTAIR | 0.4202 | 0.0041 |
| VMAC | HOTAIR | 0.4515 | 0.0018 |
| VRK2 | HOTAIR | 0.3659 | 0.0134 |
| ZEB2 | HOTAIR | -0.3830 | 0.0094 |
| ABCA8 | LINC00208 | 0.5294 | 0.0002 |
| ABCA9 | LINC00208 | 0.5421 | 0.0001 |
| ABCD2 | LINC00208 | 0.5445 | 0.0001 |
| ARHGEF35 | LINC00208 | -0.3707 | 0.0122 |
| BACH2 | LINC00208 | 0.6236 | 0.0000 |
| CARD6 | LINC00208 | 0.4451 | 0.0022 |
| CHRNA7 | LINC00208 | 0.5034 | 0.0004 |
| CNTN1 | LINC00208 | 0.6967 | 0.0000 |
| DBX2 | LINC00208 | 0.6005 | 0.0000 |
| DNAJB1 | LINC00208 | -0.4450 | 0.0022 |
| EPCAM | LINC00208 | -0.3216 | 0.0312 |
| F11R | LINC00208 | -0.4951 | 0.0005 |
| FAM32A | LINC00208 | -0.4834 | 0.0008 |
| FMN2 | LINC00208 | 0.4200 | 0.0041 |
| GABRA6 | LINC00208 | 0.7093 | 0.0000 |
| GPD1 | LINC00208 | 0.4433 | 0.0023 |
| JAM2 | LINC00208 | 0.4886 | 0.0007 |
| KANSL2 | LINC00208 | -0.3992 | 0.0066 |
| KCNA1 | LINC00208 | 0.6341 | 0.0000 |
| KRT73 | LINC00208 | 0.3144 | 0.0354 |
| MED26 | LINC00208 | -0.3336 | 0.0251 |
| MS4A12 | LINC00208 | 0.3600 | 0.0151 |
| MTX1 | LINC00208 | -0.3404 | 0.0221 |
| MYBPC1 | LINC00208 | 0.5108 | 0.0003 |
| NNAT | LINC00208 | 0.5593 | 0.0001 |
| PDCL3 | LINC00208 | -0.3042 | 0.0422 |
| PEX13 | LINC00208 | -0.3134 | 0.0361 |
| RAB25 | LINC00208 | -0.4436 | 0.0023 |
| RASEF | LINC00208 | -0.3591 | 0.0154 |
| RASL10A | LINC00208 | 0.4892 | 0.0007 |
| RNASE11 | LINC00208 | 0.6083 | 0.0000 |
| RNPEP | LINC00208 | -0.6584 | 0.0000 |
| SEMA6D | LINC00208 | 0.6413 | 0.0000 |
| SLC33A1 | LINC00208 | -0.4462 | 0.0021 |
| SYNE1 | LINC00208 | 0.5808 | 0.0000 |
| TECPR2 | LINC00208 | 0.5328 | 0.0002 |
| TIMMDC1 | LINC00208 | -0.4117 | 0.0050 |
| TLL1 | LINC00208 | 0.6772 | 0.0000 |
| TP63 | LINC00208 | 0.5246 | 0.0002 |
| TRIM38 | LINC00208 | -0.4140 | 0.0047 |
| TTC30A | LINC00208 | -0.3640 | 0.0140 |
| VMAC | LINC00208 | -0.4877 | 0.0007 |
| VRK2 | LINC00208 | -0.3032 | 0.0429 |
| WSCD2 | LINC00208 | 0.4436 | 0.0023 |
| ZEB2 | LINC00208 | 0.4813 | 0.0008 |
| ABCA8 | MEG3 | 0.4417 | 0.0024 |
| ABCA9 | MEG3 | 0.4520 | 0.0018 |
| ABCD2 | MEG3 | 0.5564 | 0.0001 |
| ARHGEF35 | MEG3 | -0.5791 | 0.0000 |
| BACH2 | MEG3 | 0.4955 | 0.0005 |
| C7orf25 | MEG3 | -0.3386 | 0.0229 |
| CD46 | MEG3 | -0.3074 | 0.0400 |
| CHMP4C | MEG3 | -0.4704 | 0.0011 |
| CHN1 | MEG3 | 0.5588 | 0.0001 |
| CHRNA7 | MEG3 | 0.3280 | 0.0278 |
| CHUK | MEG3 | -0.4032 | 0.0060 |
| CNTN1 | MEG3 | 0.7791 | 0.0000 |
| DBX2 | MEG3 | 0.5847 | 0.0000 |
| DNAJB1 | MEG3 | -0.5654 | 0.0001 |
| EPCAM | MEG3 | -0.4833 | 0.0008 |
| F11R | MEG3 | -0.5729 | 0.0000 |
| FAM32A | MEG3 | -0.6218 | 0.0000 |
| FAM83H | MEG3 | -0.3391 | 0.0227 |
| FMN2 | MEG3 | 0.5469 | 0.0001 |
| GABRA6 | MEG3 | 0.7552 | 0.0000 |
| GPD1 | MEG3 | 0.4070 | 0.0055 |
| JAM2 | MEG3 | 0.5977 | 0.0000 |
| KANSL2 | MEG3 | -0.3896 | 0.0082 |
| KCNA1 | MEG3 | 0.8326 | 0.0000 |
| MED26 | MEG3 | -0.3690 | 0.0126 |
| MPZL1 | MEG3 | -0.4313 | 0.0031 |
| MYBPC1 | MEG3 | 0.4918 | 0.0006 |
| MYNN | MEG3 | -0.3055 | 0.0413 |
| NNAT | MEG3 | 0.7656 | 0.0000 |
| PDCL3 | MEG3 | -0.3209 | 0.0316 |
| PEX13 | MEG3 | -0.3971 | 0.0069 |
| RAB25 | MEG3 | -0.5661 | 0.0001 |
| RASEF | MEG3 | -0.4163 | 0.0045 |
| RASL10A | MEG3 | 0.4953 | 0.0005 |
| RNASE11 | MEG3 | 0.3632 | 0.0142 |
| RNPEP | MEG3 | -0.4231 | 0.0038 |
| SEMA6D | MEG3 | 0.5277 | 0.0002 |
| SLC33A1 | MEG3 | -0.5287 | 0.0002 |
| SLK | MEG3 | -0.3143 | 0.0355 |
| SYNE1 | MEG3 | 0.6054 | 0.0000 |
| TECPR2 | MEG3 | 0.6289 | 0.0000 |
| TIMMDC1 | MEG3 | -0.4020 | 0.0062 |
| TLL1 | MEG3 | 0.6540 | 0.0000 |
| TMCO1 | MEG3 | -0.3369 | 0.0236 |
| TP63 | MEG3 | 0.3004 | 0.0450 |
| TRIM38 | MEG3 | -0.5464 | 0.0001 |
| TTC30A | MEG3 | -0.3019 | 0.0439 |
| VMAC | MEG3 | -0.3442 | 0.0206 |
| VRK2 | MEG3 | -0.4264 | 0.0035 |
| WSCD2 | MEG3 | 0.5566 | 0.0001 |
| ZEB2 | MEG3 | 0.4149 | 0.0046 |
| ABCA8 | THAP7-AS1 | 0.3111 | 0.0375 |
| BACH2 | THAP7-AS1 | 0.3435 | 0.0209 |
| CHMP4C | THAP7-AS1 | 0.3574 | 0.0159 |
| CREB3 | THAP7-AS1 | 0.3013 | 0.0443 |
| DNAJB1 | THAP7-AS1 | -0.3081 | 0.0395 |
| GORASP1 | THAP7-AS1 | 0.3268 | 0.0284 |
| MS4A12 | THAP7-AS1 | 0.3685 | 0.0128 |
| MYBPC1 | THAP7-AS1 | 0.4221 | 0.0039 |
| RNASE11 | THAP7-AS1 | 0.3766 | 0.0108 |
| SEMA6D | THAP7-AS1 | 0.3915 | 0.0078 |
| SLK | THAP7-AS1 | -0.3537 | 0.0171 |
| TDRD5 | THAP7-AS1 | 0.3759 | 0.0109 |
| TP63 | THAP7-AS1 | 0.3477 | 0.0193 |
| CCDC105 | TTC28-AS1 | 0.4787 | 0.0009 |
| CHRNA7 | TTC28-AS1 | 0.3431 | 0.0210 |
| CIITA | TTC28-AS1 | 0.3015 | 0.0441 |
| GNGT2 | TTC28-AS1 | 0.3017 | 0.0440 |
| GORASP1 | TTC28-AS1 | 0.4131 | 0.0048 |
| HNF4G | TTC28-AS1 | 0.4901 | 0.0006 |
| KRT73 | TTC28-AS1 | 0.7126 | 0.0000 |
| KRT75 | TTC28-AS1 | 0.6573 | 0.0000 |
| KRT78 | TTC28-AS1 | 0.3103 | 0.0380 |
| MPZL1 | TTC28-AS1 | 0.3539 | 0.0171 |
| MS4A12 | TTC28-AS1 | 0.5674 | 0.0000 |
| MYNN | TTC28-AS1 | 0.4062 | 0.0056 |
| OIT3 | TTC28-AS1 | 0.8753 | 0.0000 |
| OTOL1 | TTC28-AS1 | 0.6510 | 0.0000 |
